# Using social media to promote academic research: Identifying the benefits of twitter for sharing academic work

**DOI:** 10.1371/journal.pone.0229446

**Published:** 2020-04-06

**Authors:** Samara Klar, Yanna Krupnikov, John Barry Ryan, Kathleen Searles, Yotam Shmargad

**Affiliations:** 1 School of Government & Public Policy, University of Arizona, Tucson, AZ, United States of America; 2 Department of Political Science, Stony Brook University, Stony Brook, NY, United States of America; 3 Manship School of Mass Communication & Department of Political Science, Louisiana State University, Baton Rouge, LA, United States of America; 4 School of Government & Public Policy, University of Arizona, Tucson, AZ, United States of America; West Pomeranian University of Technology, POLAND

## Abstract

To disseminate research, scholars once relied on university media services or journal press releases, but today any academic can turn to Twitter to share their published work with a broader audience. The possibility that scholars can *push* their research out, rather than hope that it is *pulled* in, holds the potential for scholars to draw wide attention to their research. In this manuscript, we examine whether there are systematic differences in the types of scholars who most benefit from this push model. Specifically, we investigate the extent to which there are gender differences in the dissemination of research via Twitter. We carry out our analyses by tracking tweet patterns for articles published in six journals across two fields (political science and communication), and we pair this Twitter data with demographic and educational data about the authors of the published articles, as well as article citation rates. We find considerable evidence that, overall, article citations are positively correlated with tweets about the article, and we find little evidence to suggest that author gender affects the transmission of research in this new media.

Social media provide academics with one of the most direct routes for sharing their work. To disseminate research, scholars once relied on university media services or journal press releases, but today any academic can turn to Twitter to share their published findings with a broader audience that stretches well beyond their friends, family, or even academic community. In this manuscript, we provide a broad empirical investigation of whether Twitter offers any advantage to academics who share their work via social media. We then turn to the more specific question of whether Twitter offers an equitable benefit to all academics who participate, or if instead it simply exacerbates inequalities in research dissemination that exist “off-line.” In considering these inequalities, we focus on the specific case of gender.

Relying on social media, researchers can reach practitioners, journalists, and the public at large [1,2,3,4]. Twitter seems to offer tremendous benefits [5], especially given increasing interest in making academic work broadly accessible across disciplinary boundaries, as well as to the public [6]. For example, the United Kingdom now makes the commitment to dissemination of research a function of national research funding [7]. Indeed, user patterns suggest that many scholars across the globe have started using Twitter to promote their work [8].

The benefit of Twitter (and other social media platforms) is that it shifts the dissemination of scientific research from a “pull” model to a “push” model [1]. A pull model requires people who are interested in ongoing scientific research to search through publications to obtain the information; a push model allows scholars to transmit the information more directly to potentially interested parties [1]. In the pull model, the audience must have the initiative to find scientific research; in the push model, it is the researcher who initiates communication with the audience. The possibility that scholars can *push* their research out, rather than hope that it is *pulled* in, holds the potential for scholars to draw wide attention to their research [9]. While the causal connection between pushing research articles on Twitter and article citation rates is under debate [10][11], there is evidence to suggest that shared articles do have higher download and view rates [1,12]. In general, there is some correlation between Twitter and the “uptake” of new research [13], suggesting that social media use can indeed help to promote research beyond the audience they would have enjoyed in the absence of this new technology.

## Can social media combat inequalities in the dissemination of research?

While some research suggests that social media can broaden academic [13], scholars debate whether social media replicate pre-existing structural inequalities [14] or rather to help to eliminate them. On one hand, social media might simply reward those who have more resources and status [15]. On the other hand, social media can overcome resource gaps and mitigate social inequalities across a variety of domains such as social support in communities [16], campaign contributions [17], political participation [18] and even access to medical resources [19].

Research on social media use by academics has engaged in a similar debate. While issues of digital literacy may be less focal (though potentially present) for academics [20], issues of status are still salient. Even when the goal is to obtain and disseminate research, patterns of Twitter followers still show evidence of academic hierarchies, which suggests that social media may replicate inequalities [21]. Other researchers, however, use surveys of academics who use social media to show that many perceive it to be highly beneficial for early career scholars who aim to raise their profiles [20], suggesting the potential for social media to equalize whose work gets attention. In this study, we consider this debate in the context of research dissemination. We investigate whether social media serves as an equalizing force within academia by allowing academics to disseminate their work without the traditional gatekeeping of existing academic hierarchies, or whether social media merely reinforces existing systematic biases in whose academic research tends to receive broader attention.

## Research dissemination and gender in the academy

While there are numerous factors that can affect the dissemination of and attention to research off-line, existing work finds a persistent disparity between men and women in academia. Fewer women than men are invited to give seminar talks [22] and publications by women are less likely to be included in graduate syllabi [23]. Fewer women than men submit to publish journals [24] and few women publish in them [25]. In one of the most consistent findings on gender and publication, researchers show that publications by women are cited less often in academic research [26,27,28].

Underlying these differences are patterns in authorship. Working in teams with co-authors–rather than producing solo-authored papers–appears to be more beneficial for men [24,29]. Men who co-author papers not only submit and publish more articles per year [24] but also receive more citations [30]. Women, in contrast, do not benefit from co-authorship in either productivity [24] or citation counts [30]. In sum, there is evidence to suggest that solo-authored papers by women are treated differently than co-authored papers in which women are part of the authorship team [29].

Yet much of the previous work on gender relies on the pull method: a scholarly audience finds research to cite, papers to include on syllabi, or speakers to invite to seminars. It possible, however, that gendered gatekeeping affects which research is “pulled” [22] but the same barriers may be less likely to affect what happens when the research is “pushed.” Shifting to the push method on social media can potentially eliminate some gendered gatekeeping in the dissemination of research. Bowman [31], for example, finds that women academics are as likely to be on Twitter than their male colleagues (in fact slightly *more* likely, though the difference is not statistically significant).

In our analysis, we test whether using Twitter–e.g. pushing research—differentially benefits male academics who share their work via the social media platform or if, instead, it equalizes off-line gender disparities. In conducting our analyses, we follow previous research and test not only for gender disparities generally but also for differences between solo-authored and co-authored work. In addition, we account for other qualities that might explain why some research receives more attention than others.

## Data collection procedure

We began by creating a dataset of every article published in 2016 in the following academic journals: *American Political Science Review* (impact factor: 3.316), *Journal of Communication* (impact factor: 3.973), *American Politics Research* (impact factor: 1.089), *Political Research Quarterly* (impact factor: 1.523), *Journalism and Mass Communication Quarterly* (impact factor: 1.706), and *Political Communication* (impact factor: 2.738). We selected journals based on a purposive sampling strategy: we deliberately chose a range of journals from two social science fields. We intentionally selected six different journals that cover a broad range of impact factors and thus have different standing within their respective disciplines. During the time-frame of our study, these journals published a total of 308 articles, which were collectively written by 576 authors (or 552 unique authors).

Once we had generated the dataset of articles, three different research assistants coded each article for the following information: (1) the number of authors on each article, (2) the gender of each author, (3) each author’s professional rank and title, (4) the ranking of each author’s department according to the US News & World graduate rankings, (5) each author’s number of Twitter followers, (6) the number Web of Science citations to the article (7) the number of Google scholar citations to the article, and (8) categorization of the article’s subject-matter. We include the coding guidelines in S1 Appendix. The number of citations was captured twice: first, in the initial round of coding in August of 2018, and then again nearly a year later in July 2019. There were 9 articles where we could not identify the gender of the authors and 5 articles where we could not identify the professional rank of the authors. This left us with a total of 294 articles in the final dataset. Of the total list of articles in our final dataset, 48 percent had at least one woman listed as an author.

We then searched for each article’s URL using Twitter’s Advanced Search (see http://twitter.com/search-advanced). For each tweet of an article’s URL, we collected the tweet’s ID, the handle (i.e., screen name) of the account that posted the tweet, and the number of retweets it received. Overall, we obtained 191 tweets that included a link to one of 76 unique articles. In the next step of the data collection procedure, we used Twitter’s Application Programming Interface (or API) to collect handles of the accounts that retweeted each tweet. Twitter limits such queries to 100 retweeters per tweet. This was not a concern in our setting, however, as the most popular tweet received exactly 100 retweets (with the next most popular tweet receiving 32 retweets). In addition to the API’s 100 retweeter limit, retweeter information was also unavailable if the user deleted or protected their account. Still, we were able to obtain the handles of 517 out of the possible 549 retweeters. Our dataset is publicly available in Harvard Dataverse (https://dataverse.harvard.edu/dataset.xhtml?persistentId=doi:10.7910/DVN/DLH12J).

**Fig 1:**
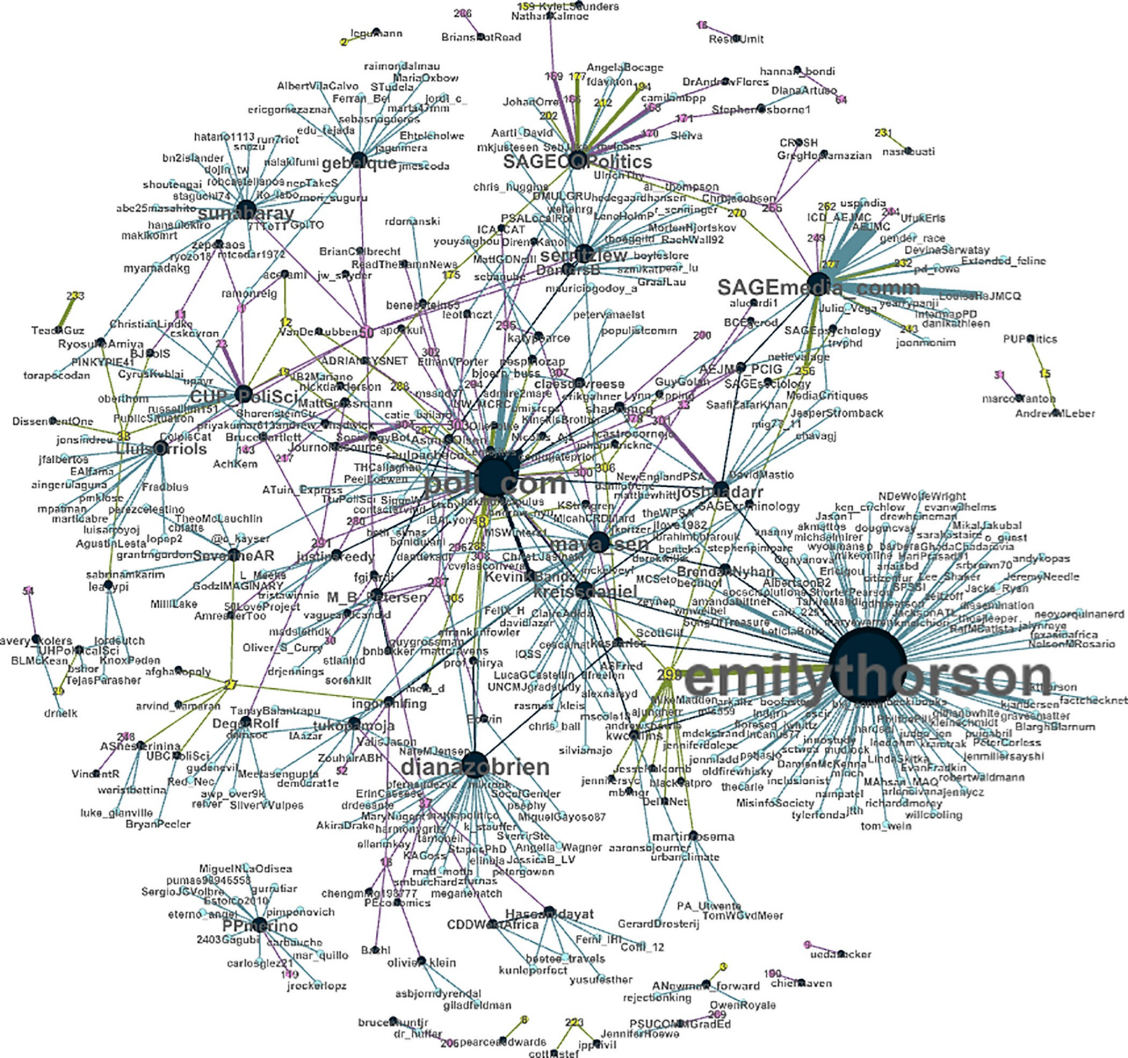
Social network of articles, tweeters, and retweeters.

In Fig 1, we depict the social network of articles, the handles that tweeted links to these articles, and the handles that retweeted these tweets. We only include the 76 articles that received at least one tweet. We define edges in this network to be between an article and the handles that tweeted its URL, and between handles that tweeted out article links and those that retweeted these tweets. We color article nodes by the gender of the lead author, with pink and yellow nodes representing man- and woman-led articles, respectively. We color handle nodes by whether they were original tweeters (black) or retweeters (blue). The size of each node reflects the number of edges it has in the network. Each article is labeled with an ID number; in the S2 Appendix, we include a legend displaying the journal, year, and lead author of each article by ID number. (More variables for each article is available from the authors).

The social network in Fig 1 reveals several descriptive aspects of our data that are worth mentioning. First, even though data were collected separately for each journal article, the network appears to be well-connected. Indeed, the largest connected component includes over 70% of the articles in the network (56 out of 76 articles). This implies that some accounts tweeted out more than one article, retweeted more than one tweet about these articles, or tweeted about some articles while also retweeting tweets about others. Second, many of the largest nodes in the network are not people, but journals. Indeed, among the top ten most well-connected handles, four are journals (see Table 1 for the full list). Among the six who are individuals, there is equal distribution between men and women. Our next step, then, is to consider the patterns in Table 1 more broadly.

**Table 1:** Top tweeters of journal articles.

Twitter Handle	# of Edges	Type
emilythorson	110	Academic
poli_com	51	Journal
dianazobrien	31	Academic
SAGEmedia_comm	26	Journal
maya_sen	23	Academic
sunaharay	20	Academic
SAGECQPolitics	19	Journal
serritzlew	19	Academic
CUP_PoliSci	17	Journal
kreissdaniel	17	Academic

Given theories of “pushing” research, we conduct our analysis in two steps. First, we consider whether we observe any systematic patterns in which articles are shared on Twitter. Second, we consider whether the patterns we observe in Twitter sharing have any effect on citations (an important academic metric).

## Patterns of Twitter dissemination

Since our goal is to consider whether Twitter ameliorates or perpetuates inequalities within academia, we first consider whether there are any systematic patterns to which articles are most or least likely to be “pushed” via Twitter. Given the particular focus of this manuscript, we want to consider whether, on average, research produced by women is shared at the same rate as research produced by men. Although Fig 1 demonstrates that several of the largest nodes in the network are female Twitter users, we want to track gender patterns more systematically. Therefore, as a next step we estimate two models, each using the article as the unit of analysis. These models track the likelihood that an article is mentioned on Twitter, using author and journal characteristics as predictors.

We begin with the models that estimate Twitter mentions, relying on two different outcome measures. The first outcome measure we use is the number of *originating* Tweets an article received (i.e. the number of Twitter users who individually tweeted about a given article). The second model uses the *total* number of tweets an article received by combining originating tweets with the total number of re-tweets they received (i.e. the total number of mentions for an article). This outcome variable captures both re-tweets in which a user added no additional content, as well as re-tweets in which a user included the original tweet along with their own message (“quote tweets”). For example, if two separate Twitter users individually tweeted about the same research article, this would mean a count of 2 in our model. If each of these two initial tweets was then retweeted 5 times, this would mean a count of 12 in our model: 10 retweets (combining both initial tweets) and 2 original tweets. What further differentiates this outcome variable from the network analyses that produced Fig 1 is that now we use all the articles in our sample, rather than only the articles that received tweets. This means that in the models that follow, a number of articles have a score of 0, since they were never tweeted at all.

Both the originating tweets model and total tweets model include the same sets of explanatory variables. First, to capture the role of author characteristics in sharing, we include the following measures: (1) the percentage of the authors on an article who are women *(% Women*), (2) the total number of authors on an article (*Number of Authors*), and (3) the interaction of these two measures. We interact the two variables to determine if there are different gender dynamics when papers are solo-authored versus co-authored following previous research (e.g. [29,24]). Alongside this set of measures capturing author characteristics, we also include the logged number of Twitter followers the authors have (in cases of multiple authors on an article, these are combined across all authors); we log this number because the effect of each additional Twitter follower should diminish as the total number of followers increases. Finally, we also include the mean academic rank across all of the authors on a given article. (In the S3 Appendix, we rerun all of these analyses but replace the percentage of women with a dummy indicator for whether the lead author is a woman. The results are substantively no different from those that we present here.)

In addition to measures capturing various author characteristics, our explanatory variables also account for potential differences in article content and potential differences across journal. To control for article content, we include dummy variables for the various defined subfields within the disciplines whose journals we are analyzing (field coding information in S1 Appendix). We also include dummy variables for each journal.

As our dependent variables are counts of tweets (either originating or total), a Poisson distribution would initially seem to be an appropriate approach to measurement. A plot of the data, however, demonstrates an overdispersion problem (see S4 Appendix). Therefore, we estimate negative binomial models in Table 2. The first column of Table 2 presents the results of a model that use the count of originating tweets as an outcome variable, which ranges from 0 to 14 (Model 1). The second column presents the model that uses total number of tweets as the outcome of interest; this variable ranges from 0 to 143 (Model 2).

**Table 2:** Predicting the number of tweets by article characteristics.

		Model 1: Originating Tweets			Model 2: Total Tweets		
		Coef.	S.E.	Z	Coef.	S.E.	Z
*Author Information*							
	% Woman	1.587	0.623	2.55	2.846	0.780	3.65
	Number of Authors	0.278	0.159	1.75	0.917	0.199	4.6
	% Women X Number of Authors	-0.491	0.343	-1.43	-1.059	0.405	-2.61
	Logged Twitter Followers	0.076	0.037	2.04	0.115	0.051	2.24
	Mean Academic Rank of Authors	0.417	0.228	1.83	0.209	0.299	0.7
*Article Subfield*							
	International Relations	-0.089	0.905	-0.1	-0.124	1.261	-0.1
	Comparative Politics	0.311	0.821	0.38	-0.020	1.135	-0.02
	Political Philosophy	-0.212	0.927	-0.23	0.040	1.272	0.03
	American Politics	0.223	0.847	0.26	0.221	1.188	0.19
	Communications	0.770	1.015	0.76	1.428	1.477	0.97
*Journal*							
	Journal of Communication	-1.103	1.211	-0.91	-0.970	1.406	-0.69
	JMCQ	1.880	0.972	1.93	2.465	1.283	1.92
	Political Communication	3.866	0.783	4.93	5.311	0.914	5.81
	Political Research Quarterly	2.225	0.792	2.81	2.620	0.910	2.88
	APSR	3.121	0.809	3.86	4.159	0.967	4.3
	Constant	-5.386	1.301	-4.14	-6.431	1.707	-3.77
	A	1.301	0.344		4.027	0.675	
N = 294							

*Negative-Binomial models*. *Dependent variable in Model 1 is originating tweets; the dependent variable in Model 2 is total tweets (originating tweets and re-tweets)*. *American Politics Research is the excluded journal category*.

Turning first to the results of Model 1, which relies on originating tweets only, we first see that the interaction between *% Women* and *Number of Authors* does not reach conventional levels of statistical significance. Nonetheless, as the statistical significance of the coefficient on an interaction may not always be indicative of a lack of effect at all levels of a moderator, we also consider the marginal effects of the *% Women* variable for various levels of *Number of Authors*. Here, we observe that the marginal effect of increases in the *% Women* variable only reaches conventional levels of significance (*p<0*.*05*) when the articles are solo-authored. Here, articles authored by one male author receive, on average, 0.6 fewer initial tweets than articles authored by a solo female author.

**Fig 2:**
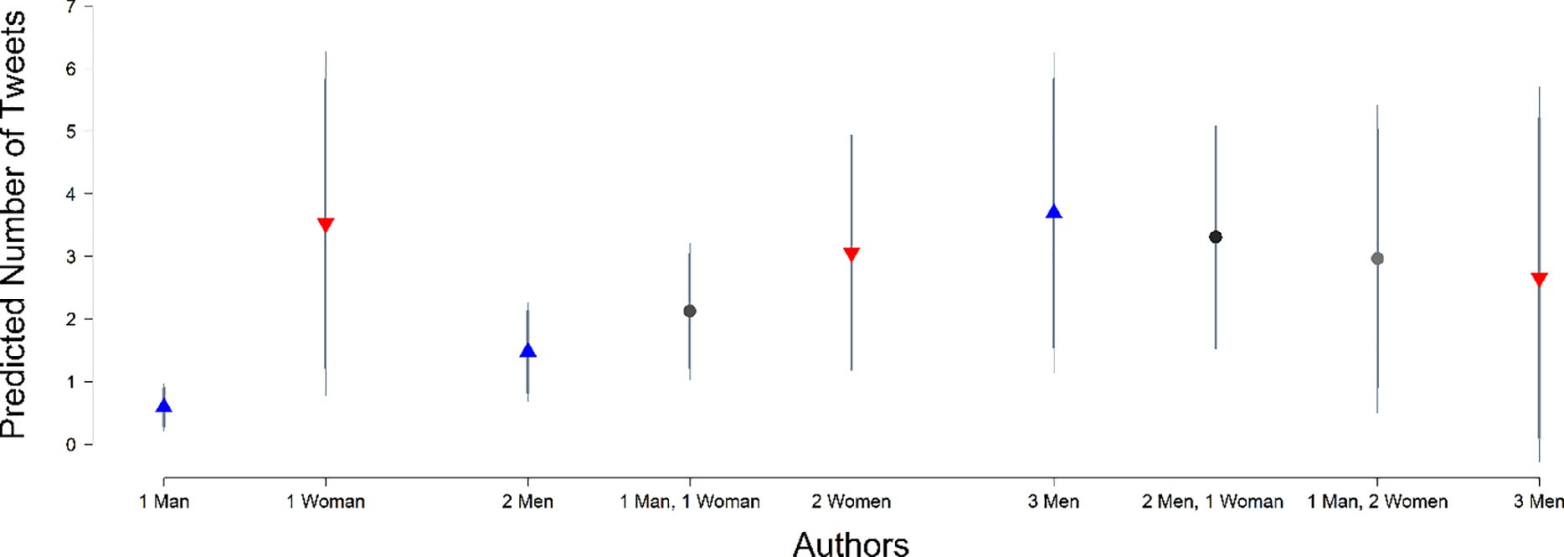
Expected number of total tweets by author gender. Figure based on estimates in Model 2, Table 2. Thinner lines represent 95% confidence interval, thicker lines 90% confidence interval around estimate. Categories are possible gender combinations of authors on published articles.

In Model 2 we track the total number of tweets (both originating tweets and re-tweets). When we shift to this outcome measure, we observe a statistically significant interaction between *% Women* and *Number of Authors*. In Fig 2, we plot the predicted number of tweets for the different possible gender compositions for articles that have 1 2, or 3 authors. We note that the patterns across various author and gender combinations suggest similar patterns for Twitter sharing of articles, with one exception: much as we observed in Model 1, papers written by solo-authors receive fewer total tweets. In sum, regardless of whether we consider originating tweets only or total tweets, we see no evidence of a gender bias in the number of tweets received by the women authors in the journals in our sample.

It is possible, however, that while women and men received similar numbers of tweets, the tweets differ in content. Perhaps, for example, men received tweets that were more positive in tone than women. To consider this possibility the we code the content of original tweets and re-tweets where the re-tweeting user added their own text (“quote tweets”). We see no evidence of differences in content. The bulk of the tweets both, men and women receive are promotional in nature–i.e. simply noting that the article has been published–and positive. Our coding scheme and gender comparisons of content are in S5A and S5B Appendix.

## Do observed Twitter patterns have any effect on citations?

To this point, our data suggest little evidence that women are less likely to have their research shared–“pushed”—on Twitter. Rather, we see some evidence that solo-authored articles by women are more likely to be shared than are solo-authored articles by men. As a next step, we then consider if and how these patterns in sharing on Twitter are associated with scholarly citations. Specifically, we investigate whether research that is “pushed” via Twitter benefits in greater citations over time, relative those that were not. Moreover, considering citations offers us an additional approach to considering the relationship between research dissemination and gender.

As we discuss earlier, we collected our initial data on Twitter sharing patterns in June 2018. In addition to the patterns in tweets, we also collected the number of citations for each of the articles in our sample at two different points in time: August 2018 (soon after the tweets) and July 2019 (one year later). The number of citations is from the Web of Science. We were unable to find the number of citations for one of the articles in 2018. We note that there is a relationship between these two time points: articles that had more citations in August 2018 continued to have more citations in July 2019 (see S6 Appendix). This is consistent with previous research demonstrating that early popularity increases subsequent popularity [32,33]. Using this citation data, we consider the relationship between tweets and citations more generally, before turning to the role of gender in this relationship.

### Tweets and citations, without gender

In Table 3, we again use Negative-Binomial models. The dependent variable in Model 1 is the number of citations in 2018. The dependent variable in Models 2 and 3 are the number of citations in 2019. In Model 3, we include the number of 2018 citations as a control variable. The number of 2018 citations ranges from 0 to 21 with 26% of articles having 0 citations. The number of 2019 citations ranges from 0 to 40 with 18% of articles having 0 citations.

**Table 3:** Predicting the number of citations by article characteristics and tweets.

		Model 1: 2018 Citations		Model 2: 2019 Citations		Model 3: 2019 Citations	
		Coef.	S.E.	Coef.	S.E.	Coef.	S.E.
	Citations 2018					0.201	0.010
	Any Tweets	0.621	0.167	0.642	0.165	-0.052	0.095
	Total Tweets	0.004	0.006	0.009	0.005	0.006	0.002
*Author Information*							
	% of Women	-0.340	0.353	-0.706	0.347	-0.822	0.196
	Number of Authors	0.054	0.091	0.000	0.094	-0.102	0.048
	% Women X Number of Authors	0.258	0.192	0.434	0.191	0.445	0.099
	Logged Twitter Followers	0.032	0.020	0.033	0.020	0.017	0.011
	Mean Academic Rank of Authors	0.070	0.099	0.040	0.095	0.025	0.054
*Article Subfield*							
	International Relations	-0.364	0.571	-0.526	0.577	-0.125	0.269
	Comparative Politics	-0.019	0.537	-0.152	0.548	-0.145	0.249
	Political Philosophy	-0.933	0.585	-1.163	0.583	-0.599	0.284
	American Politics	-0.076	0.543	-0.408	0.548	-0.356	0.252
	Communications	-0.323	0.601	-0.581	0.610	-0.412	0.297
*Journal*							
	Journal of Communication	0.540	0.479	0.617	0.511	0.256	0.268
	JMCQ	0.532	0.463	0.564	0.496	0.210	0.257
	Political Communication	0.217	0.290	0.177	0.285	0.214	0.167
	Political Research Quarterly	0.125	0.259	0.195	0.253	0.127	0.150
	APSR	0.753	0.289	0.813	0.281	0.313	0.164
	Constant	0.275	0.681	1.223	0.679	0.923	0.332
A		0.749	0.104	0.853	0.095	0.102	0.027
AIC		1312.1		1614.7		1315.6	
N		293		294		293	

All models are estimated using Negative-Binomial models. The dependent variable in Model 1 is the number of citations in 2018; the dependents variables in Models 2 and 3 are citations in 2019. The difference between Models 2 and 3 is the inclusion of a control for tweets in 2018. The difference in N between Model 2 and Models 1, 3 is that in the original coding the number of citations for one article could not be found, but was found in 2019. The Any Tweets variable is coded if an article received any tweets at all, 0 otherwise. The total tweets variable is the number of originating tweets and re-tweets (the outcome variable from Model 2 in Table 2).

To see if the number of tweets an article receives correlates with citations, we include two variables. The first, *Any Tweets*, is a dummy variable coded 1 if the article received any tweets at all and 0 if the article received no tweets. Next, *Number of Tweets* is the dependent variable from Model 2 in Table 2 –it is the total number of tweets and retweets. This allows us to see if there is any impact of an article receiving any exposure on Twitter and then to see if it matters how much exposure the article receives.

**Fig 3:**
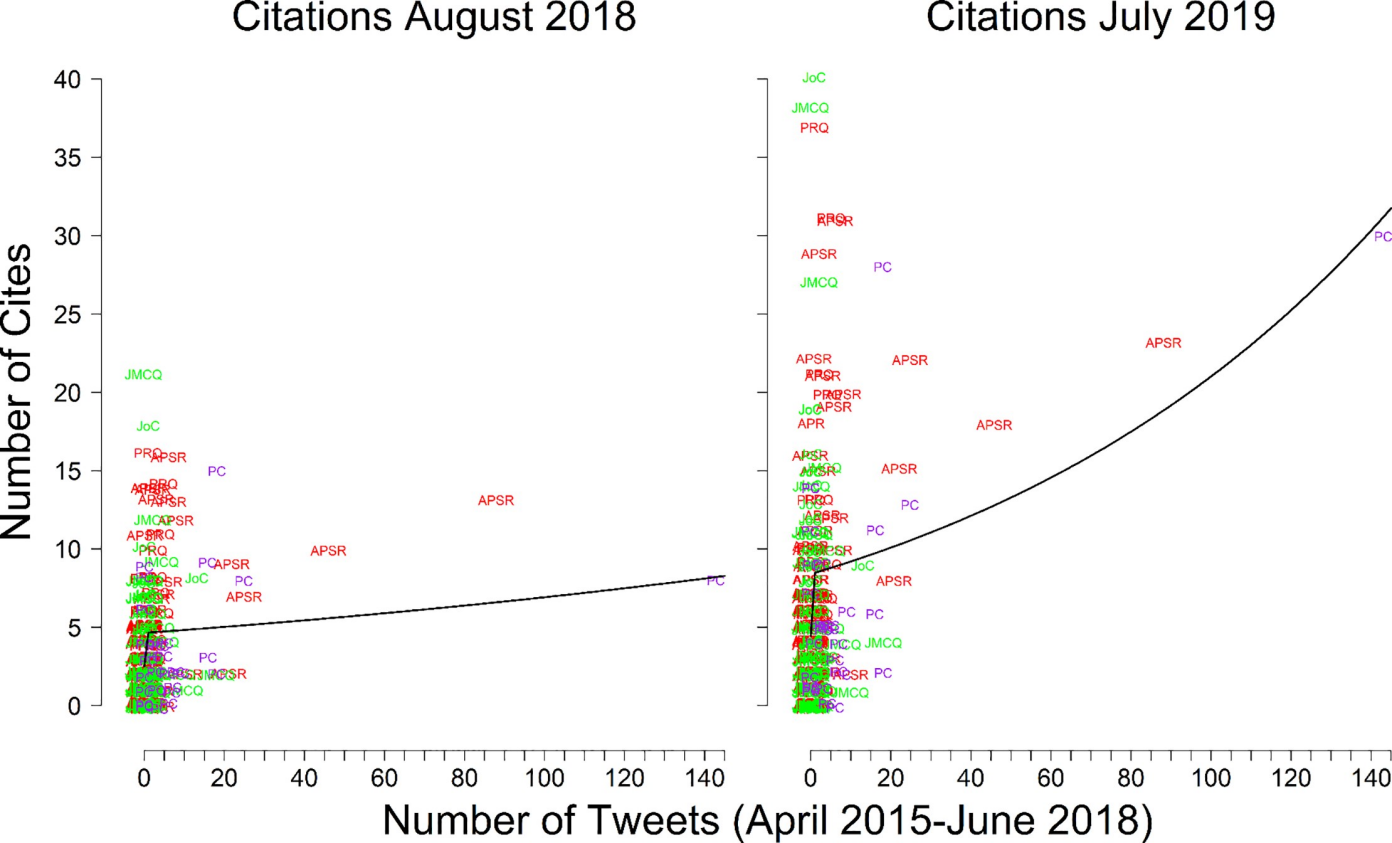
Comparing tweets and citation patterns (predictions from Model 1 and Model 2 in Table 3). Points are values represented by journal name. Black line is the predicted regression line. Both parts of the figure show an outlier at an influence point. When the outlier is removed, then only the “any tweet” variable is statistically significant–having any tweets gets leads to 4 more citations.

We plot the predicted citations by the number of tweets from Table 3‘s Model 1 (2018 citations) and Model 2 (2019 citations) in Fig 3. Each point in the plot is an article’s total tweets and citations. The black line is the predicted regression line from the model. When we consider the August 2018 citations (Model 1, Table 3), we find that the initial tweets are positively correlated, but subsequent tweets are not. In other words, receiving an initial tweet had a positive effect on citations, but each additional re-tweet did not have a linear effect on citation counts. This changes in the July 2019 model (Model 2, Table 3), which shows continued growth in citations as the number of tweets increases. Tracking the substantive effects from Model 3 tells a similar story. Even once we control for the lagged number of citations, articles with more tweets receive more citations.

We note, however, that there is one article in our sample that is a major influence point. This article has both the most tweets and more citations than all but a few articles. In S7 Appendix, we reanalyze our data excluding this outlying article. Excluding this outlier leads to results that remain similar to what we find in Model 1 (Table 3). When we leave the outlier article out of the analysis, there is still a difference between articles that receive no tweets and articles that receive at least one tweet, and again, very little effect of receiving subsequent tweets and re-tweets. Articles that received even one initial tweet received 4 more citations that those that received no tweets. However, having 50 tweets results in only one half more citations than 1 tweet.

### Tweets and citations, accounting for gender

As our goal in this manuscript is to consider whether there are gender differences in the extent to which articles are shared on Twitter, we next consider whether gender affects citation patterns. In S8 Appendix, we model the effect of author gender on citations without controlling for the number of tweets. There we see that the *% Women* variable is not statistically significant in any model and there is no interaction between gender and the number of authors in any model. This is consistent with the results in Table 3, Model 1, that do include the number of tweets alongside the gender measures.

**Fig 4:**
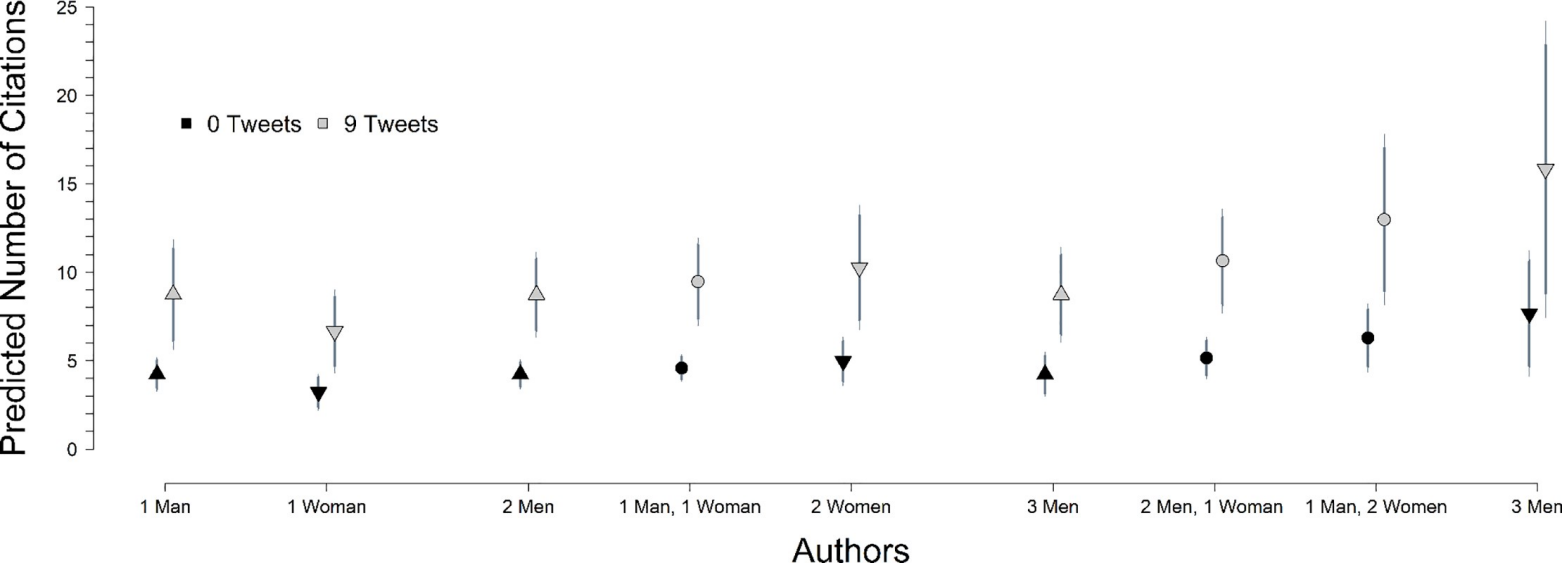
Gender of authors and citations. Predictions from Model 2 in Table 3. *Predictions in figure conditional on receiving any tweets at all*. *When we model citations without tweets*, *we see no gender effects at all; instead citations increase as number of authors increase*. *Darker lines are 90% confidence intervals*, *thinner lines are 95% confidence intervals*.

In Table 3, Models 2 and 3, however, we do see statistically significant gender differences once we control for the number of tweets an article received. We plot these results in Fig 4. There, we see that as the percentage of women authors increases, the number of citations increases but only for papers with at least 3 authors. But the figure also shows that these effects are small compared to the effect of the number of tweets the article receives in the first place. In sum, when we consider citations–rather than just tweets–we do not find any evidence of gendered dissemination patterns.

That we observe few gender differences in citations may, initially, seem incongruous with previous research suggesting that women are less likely to be cited [28,26]. There are likely several reasons for this difference. First, the prior research considered citation patterns specifically in the political science fields of international relations [26] and methodology [28], as well as in economics journals [28]. Like the previous research, we include the *American Political Science Review* in our dataset, but we also include different journals in political science and we consider journals in communication as well.

A second important difference is that the previous research considers the gender of the authors cited in an article, while we consider the number of times an article was cited by others. Given that all of our papers are relatively recent, they all have generally lower levels of citations. It is possible for example, that gender differences emerge later on in an article’s lifecycle, which our data would not capture. On the other hand, it is also possible that gender disparities are more likely to emerge for older articles–rather than papers published more recently. One additional consideration is that research producing null results is less likely to be published (Franco, Malhotra and Simonovits [34]), which could in part explain the lack of other work showing this same pattern.

## Conclusion

Twitter offers scholars the opportunities to “push” their research, rather than depending on other people to “pull” it from academic publications. Across all scholars, we find evidence that pushing research on Twitter is correlated with greater citation counts. Given gender disparities in other forms of academic research dissemination [28,22], we consider whether the same gender disparities exist on Twitter. Tracking newly published papers across journals with a wide range of impact factors, we find no evidence that more women authors on an article decrease Twitter sharing. Rather, we find that articles that are least likely to be shared on Twitter are authored by a solo man. Further, we also do not see any additional gender disparities in the relationship between these tweets and eventual article citations.

There are some limitations to our findings. First, a team of coders determined the author of each gender to the best of their ability, though it is possible that authors’ names and appearances (to the extent that photos were available online) did not perfectly inform their coding. Although we have included general journals in our sample (e.g. *American Political Science Review*), it is possible that we have focused on research areas (e.g. communication and political communication) where gender disparities are lower than they are in other research areas in disciplines (e.g. quantitative methodology, economics [28]. Indeed, there is some evidence that the political communication subfield of political science has an above average representation of women [35]. We do note, however, that our models control for the subfield of the article to account for these differences. Another limitation may be that we tracked already published articles, rather than beginning with articles that appear in First View. This may mean that some papers in our sample may have received no tweets upon publication because they received tweets when they initial appeared online *prior to publication*. While this may attenuate the number of tweets per article in our sample, it is not clear why these patterns would be gendered. In other words, while First View may have led to more articles with 0 tweets, it is unlikely that the lack of gender bias we observe is due to this effect.

## Supporting information

S1 Appendix Using social media to promote academic research. (DOCX)

S2 Appendix Legend of articles displayed in Fig 1. (DOCX)

S3 Appendix Predicting the number of tweets by article characteristics: Using “woman lead author” in place of “percent authors women”. (DOCX)

S4 Appendix Distribution of counts of tweets. (DOCX)

S5 Appendix a. Tweet content coding. b. Results of content coding, by author gender.
(DOCX)

S6 Appendix Relationship between Citations in 2018 and 2019. The figure includes some jitter because there are multiple articles with 0 citations at both time points.
(DOCX)

S7 Appendix Predicting the number of citations by article characteristics and tweets, excluding outlier. (DOCX)

S8 Appendix Modelling the effect of author gender on citations without controlling for the number of tweets. (DOCX)

S1 File (PDF)

S2 File (PDF)

S3 File (PDF)

S4 File (PDF)

S5 File (PDF)

S6 File (PDF)

S7 File (PDF)
