# Supplementary material for: Using social media to promote academic research: Identifying the benefits of twitter for sharing academic work
Source: PLoS One. 2020 Apr 6;15(4):e0229446. doi: 10.1371/journal.pone.0229446 (PMC7135289; doi:10.1371/journal.pone.0229446)
Supplement: S1 Appendix — (DOCX) [file pone.0229446.s001.docx]

**S1 Appendix. Using Social Media to Promote Academic Research.**

**A1. Coding Instrument, Articles**

We provided the following information to graduate student coders (students in political science and communication) who did not know the goals of this project.

**Article Information**

1. What is your best characterization of the dominant topical area of this article? [for political science use the following categories: international relations, comparative, theory, American politics; for communication use: communication studies, journalism, comSHER which includes science health and environmental com, and strategic communication]
   1. 0 – Methodological (Political Science)
   2. 1 – IR
   3. 2 – Comparative Politics
   4. 3 – Theory/Political Philosophy
   5. 4 – American Politics
   6. 5 – communication studies
   7. 6 – journalism
   8. 7 – comSHER
   9. 8 – strategic communication

*Note: communication categories (5-8) were all combined as there were not enough articles in some categories.*

1. How many Web of Science cites does this article have?
   1. Write in cited by number.
   2. 99–no Web of Science entry

*Note: there was one article with no Web of Science entry in 2018; we believe this was an error as it appeared during the 2019 coding.*

***Author Information***

1. [Coded for each author on the paper] What is this person’s gender?
   1. 1 – Female
   2. 2 – Male
   3. 99 – cannot identify
2. [Coded for each author on the paper] Does this person have a twitter account?
   1. Write in twitter handle
   2. 1 – no twitter handle
3. [Coded for each author on the paper] How many followers does the account have?
4. [Coded for each author on the paper] What is the person’s academic position:
   1. 1 – Grad student
   2. 2 – Post-Doc/Fellow
   3. 3 – Visiting Assistant Professor/Adjunct
   4. 4 – Assistant Professor/Lecturer (if not in the US)
   5. 5 – Associate Professor/Senior Lecturer (if not in the US)
   6. 6 – Full Professor
   7. 7 - Private Sector
   8. 99 – cannot identify

*Next set of variables are used in robustness checks:*

1. [Coded for each author on the paper] Year they obtained their Ph.D.?
   1. [year]
   2. 88 - In progress
   3. 99 – cannot identify
2. [Coded for each author on the paper] What is their current department?
   1. 1 – political science/government (or political science adjacent – public policy school)
   2. 2 – communication studies (or adjacent)
   3. 3 – other
   4. 99 – cannot identify
3. [Coded for each author on the paper] What is this person’s current affiliation?
   1. [Write in institution name]
   2. 99 – cannot identify
4. [Coded for each author on the paper] Rank of the university graduate department (for authors in political science departments we use [US News & World grad rankings](file:///C:\Users\klar\Dropbox\Twitter%20Paper\political%20science%20is%20here:%20https:\www.usnews.com\best-graduate-schools\search%3fprogram=top-political-science-schools&name=%5d) for that dept; for authors in communications studies and adjacent use Top University Rankins. Note schools past 50 for the latter are grouped, e.g. 51-100, 101-200, etc, in such cases uses the first number in the range so 51, 101, etc)
   1. Rank number
   2. 99 – cannot identify
