# Supplementary material for: Using social media to promote academic research: Identifying the benefits of twitter for sharing academic work
Source: PLoS One. 2020 Apr 6;15(4):e0229446. doi: 10.1371/journal.pone.0229446 (PMC7135289; doi:10.1371/journal.pone.0229446)
Supplement: S2 Appendix — (DOCX) [file pone.0229446.s002.docx]

# **S2 Appendix. Legend of Articles Displayed in Fig 1.**

| **ID** | **Journal** | **Year** | **Last name of Lead Author** |
| --- | --- | --- | --- |
| 1 | The American Political Science Review | 2016 | BLATTMAN |
| 2 | The American Political Science Review | 2016 | VALDEZ |
| 3 | The American Political Science Review | 2016 | JOHNS |
| 4 | The American Political Science Review | 2016 | DAVENPORT |
| 5 | The American Political Science Review | 2016 | HIRSCH |
| 6 | The American Political Science Review | 2016 | RITTER |
| 7 | The American Political Science Review | 2016 | KAPUST |
| 8 | The American Political Science Review | 2016 | O'BRIEN |
| 9 | The American Political Science Review | 2016 | BRAUN |
| 10 | The American Political Science Review | 2016 | NILI |
| 11 | The American Political Science Review | 2016 | LARREGUY |
| 12 | The American Political Science Review | 2016 | RUNDLETT |
| 13 | The American Political Science Review | 2016 | BOUSHEY |
| 14 | The American Political Science Review | 2016 | WEYLAND |
| 15 | The American Political Science Review | 2016 | HOLLAND |
| 16 | The American Political Science Review | 2016 | MURAT TEZCUR |
| 17 | The American Political Science Review | 2016 | SIGWART |
| 18 | The American Political Science Review | 2016 | BENOIT |
| 19 | The American Political Science Review | 2016 | HAN |
| 20 | The American Political Science Review | 2016 | KUYPER |
| 21 | The American Political Science Review | 2016 | ROGOWSKI |
| 22 | The American Political Science Review | 2016 | RASMUSSEN |
| 23 | The American Political Science Review | 2016 | HOLBEIN |
| 24 | The American Political Science Review | 2016 | MUNOZ |
| 25 | The American Political Science Review | 2016 | SCHWEBER |
| 26 | The American Political Science Review | 2016 | BOHMELT |
| 27 | The American Political Science Review | 2016 | SHESTERINIA |
| 28 | The American Political Science Review | 2016 | BLAIR |
| 29 | The American Political Science Review | 2016 | BAGASHKA |
| 30 | The American Political Science Review | 2016 | LAITIN |
| 31 | The American Political Science Review | 2016 | STAUFFER |
| 32 | The American Political Science Review | 2016 | CAMPBELL |
| 33 | The American Political Science Review | 2016 | ACHARYA |
| 34 | The American Political Science Review | 2016 | BRACIC |
| 35 | The American Political Science Review | 2016 | KOGANZON |
| 36 | The American Political Science Review | 2016 | FOLKE |
| 37 | The American Political Science Review | 2016 | CROKE |
| 38 | The American Political Science Review | 2016 | YPI |
| 39 | The American Political Science Review | 2016 | VALENZUELA |
| 40 | The American Political Science Review | 2016 | IMAI |
| 41 | The American Political Science Review | 2016 | VERSTEEG |
| 42 | The American Political Science Review | 2016 | ABRAMSON |
| 43 | The American Political Science Review | 2016 | CARNES |
| 44 | The American Political Science Review | 2016 | KOGELMANN |
| 45 | The American Political Science Review | 2016 | SEXTON |
| 46 | The American Political Science Review | 2016 | LEEMANN |
| 47 | The American Political Science Review | 2016 | ANZIA |
| 48 | The American Political Science Review | 2016 | CAMERON |
| 49 | The American Political Science Review | 2016 | PELED |
| 50 | The American Political Science Review | 2016 | BLOM-HANSEN |
| 51 | The American Political Science Review | 2016 | CARNES |
| 52 | The American Political Science Review | 2016 | SKARBEK |
| 53 | The American Political Science Review | 2016 | PHULWANI |
| 54 | The American Political Science Review | 2016 | MCKEAN |
| 55 | The American Political Science Review | 2016 | FRAISTAT |
| 56 | Journal of Communication | 2016 | VORDERER |
| 57 | Journal of Communication | 2016 | BOCK |
| 58 | Journal of Communication | 2016 | CHALABY |
| 59 | Journal of Communication | 2016 | EDWARDS |
| 60 | Journal of Communication | 2016 | GONZALES |
| 61 | Journal of Communication | 2016 | GRAVES |
| 62 | Journal of Communication | 2016 | NEIGER |
| 63 | Journal of Communication | 2016 | TANEJA |
| 64 | Journal of Communication | 2016 | WRIGHT |
| 65 | Journal of Communication | 2016 | POLLOCK |
| 66 | Journal of Communication | 2016 | SANTINO |
| 67 | Journal of Communication | 2016 | CAPURRO |
| 68 | Journal of Communication | 2016 | RAJANI |
| 69 | Journal of Communication | 2016 | VALENTINI |
| 70 | Journal of Communication | 2016 | GRABE |
| 71 | Journal of Communication | 2016 | KATZ |
| 72 | Journal of Communication | 2016 | BURGERS |
| 73 | Journal of Communication | 2016 | STANYER |
| 74 | Journal of Communication | 2016 | EVANS |
| 75 | Journal of Communication | 2016 | FU |
| 76 | Journal of Communication | 2016 | WESSLER |
| 77 | Journal of Communication | 2016 | EBERWEIN |
| 78 | Journal of Communication | 2016 | BOLLINGER |
| 79 | Journal of Communication | 2016 | WANG |
| 80 | Journal of Communication | 2016 | CHOULIARAKI |
| 81 | Journal of Communication | 2016 | BILLARD |
| 82 | Journal of Communication | 2016 | INCEOGLU |
| 83 | Journal of Communication | 2016 | PATTERSON |
| 84 | Journal of Communication | 2016 | BRIGHT |
| 85 | Journal of Communication | 2016 | INNOCENTI |
| 86 | Journal of Communication | 2016 | JOHNSON |
| 87 | Journal of Communication | 2016 | KOSCHMANN |
| 88 | Journal of Communication | 2016 | LEE |
| 89 | Journal of Communication | 2016 | PLAISANCE |
| 90 | Journal of Communication | 2016 | RIM |
| 91 | Journal of Communication | 2016 | VAN AELST |
| 92 | Journal of Communication | 2016 | CORREA |
| 93 | Journal of Communication | 2016 | HAENSCHEN |
| 94 | Journal of Communication | 2016 | LYNCH |
| 95 | Journal of Communication | 2016 | NATALE |
| 96 | Journal of Communication | 2016 | SALEEM |
| 97 | Journal of Communication | 2016 | RINKE |
| 98 | Journal of Communication | 2016 | TAL-OR |
| 99 | Journal of Communication | 2016 | WINTER |
| 100 | Journal of Communication | 2016 | HUANG |
| 101 | Journal of Communication | 2016 | TURNER |
| 102 | Journal of Communication | 2016 | MARTINEZ-GUILLEM |
| 103 | Journal of Communication | 2016 | DOSCH |
| 104 | Journal of Communication | 2016 | STAHL |
| 105 | Journal of Communication | 2016 | ARENDT |
| 106 | Journal of Communication | 2016 | BARTSCH |
| 107 | Journal of Communication | 2016 | BENCHERKI |
| 108 | Journal of Communication | 2016 | JACOBSON |
| 109 | Journal of Communication | 2016 | KROON |
| 110 | Journal of Communication | 2016 | LING |
| 111 | Journal of Communication | 2016 | POWERS |
| 112 | Journal of Communication | 2016 | JIA |
| 113 | Journal of Communication | 2016 | WOODWARD |
| 114 | Journal of Communication | 2016 | DIXON |
| 115 | Journal of Communication | 2016 | GUTTMAN |
| 116 | Journal of Communication | 2016 | HARWOOD |
| 117 | Journal of Communication | 2016 | MADIANOU |
| 118 | Journal of Communication | 2016 | MENG |
| 119 | Journal of Communication | 2016 | THEOCHARIS |
| 120 | Journal of Communication | 2016 | WOJCIEZAK |
| 121 | Journal of Communication | 2016 | VALENTINI |
| 122 | Journal of Communication | 2016 | THOMPSON |
| 123 | Journal of Communication | 2016 | BHATTACHARYYA |
| 124 | Journal of Communication | 2016 | GUTGOLD |
| 125 | Journal of Communication | 2016 | SEO |
| 126 | American Politics Research | 2016 | BAILEY |
| 127 | American Politics Research | 2016 | MILLER |
| 128 | American Politics Research | 2016 | CARSON |
| 129 | American Politics Research | 2016 | JEWITT |
| 130 | American Politics Research | 2016 | RUDOLPH |
| 131 | American Politics Research | 2016 | WROE |
| 132 | American Politics Research | 2016 | JOHNSTON |
| 133 | American Politics Research | 2016 | BAKER |
| 134 | American Politics Research | 2016 | HASSELL |
| 135 | American Politics Research | 2016 | HARDEN |
| 136 | American Politics Research | 2016 | BOWLER |
| 137 | American Politics Research | 2016 | RAGUSA |
| 138 | American Politics Research | 2016 | EVANS |
| 139 | American Politics Research | 2016 | PALMER |
| 140 | American Politics Research | 2016 | SIDES |
| 141 | American Politics Research | 2016 | FREEZE |
| 142 | American Politics Research | 2016 | LUTTIG |
| 143 | American Politics Research | 2016 | CAMOBRECO |
| 144 | American Politics Research | 2016 | SMITH |
| 145 | American Politics Research | 2016 | MILLER |
| 146 | American Politics Research | 2016 | DESANTE |
| 147 | American Politics Research | 2016 | HAZELTON |
| 148 | American Politics Research | 2016 | O'GREEN |
| 149 | American Politics Research | 2016 | HAZELTON |
| 150 | American Politics Research | 2016 | HALL |
| 151 | American Politics Research | 2016 | LEONARD |
| 152 | American Politics Research | 2016 | HEERSINK |
| 153 | American Politics Research | 2016 | BUTTICE |
| 154 | American Politics Research | 2016 | ROBERTS |
| 155 | American Politics Research | 2016 | MILER |
| 156 | American Politics Research | 2016 | DANCEY |
| 157 | American Politics Research | 2016 | FLYNN |
| 158 | American Politics Research | 2016 | PHINNEY |
| 159 | American Politics Research | 2016 | MILLER |
| 160 | American Politics Research | 2016 | CONDON |
| 161 | American Politics Research | 2016 | HICKS |
| 162 | American Politics Research | 2016 | DICKERSON |
| 163 | American Politics Research | 2016 | CLIFFORD |
| 164 | American Politics Research | 2016 | CALFANO |
| 165 | Political Research Quarterly | 2016 | PEKSEN |
| 166 | Political Research Quarterly | 2016 | GARRETSON |
| 167 | Political Research Quarterly | 2016 | GREENWOOD |
| 168 | Political Research Quarterly | 2016 | FLORES |
| 169 | Political Research Quarterly | 2016 | USCINSKI |
| 170 | Political Research Quarterly | 2016 | GIMBEL |
| 171 | Political Research Quarterly | 2016 | KARCH |
| 172 | Political Research Quarterly | 2016 | CASAS KLAUSEN |
| 173 | Political Research Quarterly | 2016 | ANDRE |
| 174 | Political Research Quarterly | 2016 | FARIZO MCCARTHY |
| 175 | Political Research Quarterly | 2016 | HOLMAN |
| 176 | Political Research Quarterly | 2016 | BARBER |
| 177 | Political Research Quarterly | 2016 | SANCHEZ |
| 178 | Political Research Quarterly | 2016 | MACKIN |
| 179 | Political Research Quarterly | 2016 | QUEK |
| 180 | Political Research Quarterly | 2016 | WINTERS |
| 181 | Political Research Quarterly | 2016 | CRESPIN |
| 182 | Political Research Quarterly | 2016 | BAGG |
| 183 | Political Research Quarterly | 2016 | AUDETTE |
| 184 | Political Research Quarterly | 2016 | BOWERSOX |
| 185 | Political Research Quarterly | 2016 | BUTTON |
| 186 | Political Research Quarterly | 2016 | CHANG |
| 187 | Political Research Quarterly | 2016 | JENNINGS |
| 188 | Political Research Quarterly | 2016 | GIAMARIO |
| 189 | Political Research Quarterly | 2016 | FAZEKAS |
| 190 | Political Research Quarterly | 2016 | SPRUYT |
| 191 | Political Research Quarterly | 2016 | MOREL |
| 192 | Political Research Quarterly | 2016 | RAGUSA |
| 193 | Political Research Quarterly | 2016 | GIDENGIL |
| 194 | Political Research Quarterly | 2016 | FORTIN-RITTBERGER |
| 195 | Political Research Quarterly | 2016 | OGUTCU-FU |
| 196 | Political Research Quarterly | 2016 | NEIHEISEL |
| 197 | Political Research Quarterly | 2016 | SHIN |
| 198 | Political Research Quarterly | 2016 | ANSON |
| 199 | Political Research Quarterly | 2016 | BAKER |
| 200 | Political Research Quarterly | 2016 | HOLZINGER |
| 201 | Political Research Quarterly | 2016 | COOPER |
| 202 | Political Research Quarterly | 2016 | FEEZELL |
| 203 | Political Research Quarterly | 2016 | HASSAN |
| 204 | Political Research Quarterly | 2016 | MAGALHAES |
| 205 | Political Research Quarterly | 2016 | LAPINSKI |
| 206 | Political Research Quarterly | 2016 | HUNT |
| 207 | Political Research Quarterly | 2016 | ALLEN |
| 208 | Political Research Quarterly | 2016 | ISANI |
| 209 | Political Research Quarterly | 2016 | RANDAZZO |
| 210 | Political Research Quarterly | 2016 | GARDNER |
| 211 | Political Research Quarterly | 2016 | ELLIS |
| 212 | Political Research Quarterly | 2016 | VALDEZ |
| 213 | Political Research Quarterly | 2016 | SMITH |
| 214 | Political Research Quarterly | 2016 | WALSH |
| 215 | Political Research Quarterly | 2016 | PUSOK |
| 216 | Political Research Quarterly | 2016 | HJORTH |
| 217 | Political Research Quarterly | 2016 | CHEN |
| 218 | Political Research Quarterly | 2016 | MABOUDI |
| 219 | Political Research Quarterly | 2016 | MORAL |
| 220 | Political Research Quarterly | 2016 | MADONNA |
| 221 | Political Research Quarterly | 2016 | BUEHLER |
| 222 | Political Research Quarterly | 2016 | RASMUSSEN |
| 223 | Political Research Quarterly | 2016 | BOYD |
| 224 | Political Research Quarterly | 2016 | XYDIAS |
| 225 | Political Research Quarterly | 2016 | SHIKANO |
| 226 | Political Research Quarterly | 2016 | CONNOLLY |
| 227 | Political Research Quarterly | 2016 | BUTLER |
| 228 | Political Research Quarterly | 2016 | CHATAGNIER |
| 229 | Political Research Quarterly | 2016 | VALDINI |
| 230 | Journalism and Mass Communication Quarterly | 2016 | MOY |
| 231 | Journalism and Mass Communication Quarterly | 2016 | JAHNG |
| 232 | Journalism and Mass Communication Quarterly | 2016 | APPELMAN |
| 233 | Journalism and Mass Communication Quarterly | 2016 | GUZMAN |
| 234 | Journalism and Mass Communication Quarterly | 2016 | DU |
| 235 | Journalism and Mass Communication Quarterly | 2016 | MACNAMARA |
| 236 | Journalism and Mass Communication Quarterly | 2016 | SCHERR |
| 237 | Journalism and Mass Communication Quarterly | 2016 | IRERI |
| 238 | Journalism and Mass Communication Quarterly | 2016 | SEVENANS |
| 239 | Journalism and Mass Communication Quarterly | 2016 | MIZUNO |
| 240 | Journalism and Mass Communication Quarterly | 2016 | YOUM |
| 241 | Journalism and Mass Communication Quarterly | 2016 | STOYCHEFF |
| 242 | Journalism and Mass Communication Quarterly | 2016 | STEWART |
| 243 | Journalism and Mass Communication Quarterly | 2016 | GUO |
| 244 | Journalism and Mass Communication Quarterly | 2016 | SHAHIN |
| 245 | Journalism and Mass Communication Quarterly | 2016 | SANTANA |
| 246 | Journalism and Mass Communication Quarterly | 2016 | CHEN |
| 247 | Journalism and Mass Communication Quarterly | 2016 | KIM |
| 248 | Journalism and Mass Communication Quarterly | 2016 | JOHNSON |
| 249 | Journalism and Mass Communication Quarterly | 2016 | HYUN |
| 250 | Journalism and Mass Communication Quarterly | 2016 | BOBKOWSKI |
| 251 | Journalism and Mass Communication Quarterly | 2016 | VON SIKORSKI |
| 252 | Journalism and Mass Communication Quarterly | 2016 | MAURER |
| 253 | Journalism and Mass Communication Quarterly | 2016 | CARPENTER |
| 254 | Journalism and Mass Communication Quarterly | 2016 | YEO |
| 255 | Journalism and Mass Communication Quarterly | 2016 | BUNKER |
| 256 | Journalism and Mass Communication Quarterly | 2016 | WILSON |
| 257 | Journalism and Mass Communication Quarterly | 2016 | BARD |
| 258 | Journalism and Mass Communication Quarterly | 2016 | LEE |
| 259 | Journalism and Mass Communication Quarterly | 2016 | KIM |
| 260 | Journalism and Mass Communication Quarterly | 2016 | MASSEY |
| 261 | Journalism and Mass Communication Quarterly | 2016 | CHYI |
| 262 | Journalism and Mass Communication Quarterly | 2016 | CHOI |
| 263 | Journalism and Mass Communication Quarterly | 2016 | PANEK |
| 264 | Journalism and Mass Communication Quarterly | 2016 | CHAN-OLMSTED |
| 265 | Journalism and Mass Communication Quarterly | 2016 | ZHANG |
| 266 | Journalism and Mass Communication Quarterly | 2016 | SHERRICK |
| 267 | Journalism and Mass Communication Quarterly | 2016 | CAMAJ |
| 268 | Journalism and Mass Communication Quarterly | 2016 | GINOSAR |
| 269 | Journalism and Mass Communication Quarterly | 2016 | BOWE |
| 270 | Journalism and Mass Communication Quarterly | 2016 | MORIN |
| 271 | Journalism and Mass Communication Quarterly | 2016 | PAK |
| 272 | Journalism and Mass Communication Quarterly | 2016 | YAN |
| 273 | Journalism and Mass Communication Quarterly | 2016 | MEEKS |
| 274 | Journalism and Mass Communication Quarterly | 2016 | HUTCHENS |
| 275 | Journalism and Mass Communication Quarterly | 2016 | CHEUNG |
| 276 | Journalism and Mass Communication Quarterly | 2016 | SLAVTCHEVA-PETKOVA |
| 277 | Journalism and Mass Communication Quarterly | 2016 | LOVEJOY |
| 278 | Political Communication | 2016 | ESHBAUGH-SOHA |
| 279 | Political Communication | 2016 | CARPINELLA |
| 280 | Political Communication | 2016 | REEDY |
| 281 | Political Communication | 2016 | HELFER |
| 282 | Political Communication | 2016 | TAI |
| 283 | Political Communication | 2016 | DVIR GVIRSMAN |
| 284 | Political Communication | 2016 | EYAL |
| 285 | Political Communication | 2016 | VAN DER MEER |
| 286 | Political Communication | 2016 | MASKET |
| 287 | Political Communication | 2016 | LAUSTSEN |
| 288 | Political Communication | 2016 | NEIMAN |
| 289 | Political Communication | 2016 | WOJCIEZAK |
| 290 | Political Communication | 2016 | ROBISON |
| 291 | Political Communication | 2016 | LEVENDUSKY |
| 292 | Political Communication | 2016 | SLOTHUUS |
| 293 | Political Communication | 2016 | CIUK |
| 294 | Political Communication | 2016 | KEPPLINGER |
| 295 | Political Communication | 2016 | CHALMERS |
| 296 | Political Communication | 2016 | POTTER |
| 297 | Political Communication | 2016 | MOEHLER |
| 298 | Political Communication | 2016 | SHI |
| 299 | Political Communication | 2016 | THORSON |
| 300 | Political Communication | 2016 | PORTER |
| 301 | Political Communication | 2016 | DARR |
| 302 | Political Communication | 2016 | LELKES |
| 303 | Political Communication | 2016 | KREISS |
| 304 | Political Communication | 2016 | SOROKA |
| 305 | Political Communication | 2016 | BAILARD |
| 306 | Political Communication | 2016 | SEVENANS |
| 307 | Political Communication | 2016 | LUTTIG |
| 308 | Political Communication | 2016 | BANDA |
