# Supplementary material for: Using social media to promote academic research: Identifying the benefits of twitter for sharing academic work
Source: PLoS One. 2020 Apr 6;15(4):e0229446. doi: 10.1371/journal.pone.0229446 (PMC7135289; doi:10.1371/journal.pone.0229446)
Supplement: S3 Appendix — (DOCX) [file pone.0229446.s003.docx]

# **S3 Appendix.** **Predicting the number of tweets by article characteristics: Using “Woman Lead Author” in place of “Percent Authors Women”.**

|  |  | |  | Model 1:  Originating Tweets | | |  | Model 2:  Total Tweets | | |
| --- | --- | --- | --- | --- | --- | --- | --- | --- | --- | --- |
|  |  | |  | Coef. | S.E. | Z |  | Coef. | S.E. | Z |
|  | | *Author Information* | |  |  |  |  |  |  |  |
|  |  | | Woman Lead Author | 1.926 | 0.536 | 3.59 |  | 3.321 | 0.711 | 4.67 |
|  |  | | Number of Authors | 0.404 | 0.171 | 2.37 |  | 1.056 | 0.205 | 5.15 |
|  |  | | % Women X Number of Authors | -0.653 | 0.240 | -2.71 |  | -1.297 | 0.322 | -4.03 |
|  |  | | Logged Twitter Followers | 0.079 | 0.037 | 2.15 |  | 0.123 | 0.049 | 2.50 |
|  |  | | Mean Academic Rank of Authors | 0.365 | 0.225 | 1.62 |  | 0.193 | 0.295 | 0.66 |
|  | | *Article Subfield* | |  |  |  |  |  |  |  |
|  |  | | International Relations | 0.215 | 0.905 | 0.24 |  | 0.250 | 1.21 | 0.21 |
|  |  | | Comparative Politics | 0.432 | 0.820 | 0.53 |  | 0.172 | 1.107 | 0.16 |
|  |  | | Political Philosophy | 0.063 | 0.935 | 0.07 |  | 0.453 | 1.248 | 0.36 |
|  |  | | American Politics | 0.344 | 0.847 | 0.41 |  | 0.382 | 1.154 | 0.33 |
|  |  | | Communications | 0.962 | 1.00 | 0.96 |  | 1.755 | 1.467 | 1.20 |
|  | | *Journal* | |  |  |  |  |  |  |  |
|  |  | | Journal of Communication | -1.155 | 1.197 | -0.96 |  | -1.263 | 1.419 | -0.89 |
|  |  | | JMCQ | 1.888 | 0.956 | 1.97 |  | 2.538 | 1.281 | 1.98 |
|  |  | | Political Communication | 3.983 | 0.793 | 2.81 |  | 5.453 | 0.918 | 5.94 |
|  |  | | Political Research Quarterly | 2.230 | 0.793 | 2.81 |  | 2.613 | 0.917 | 2.85 |
|  |  | | APSR | 3.094 | 0.808 | 3.83 |  | 4.110 | 0.960 | 4.28 |
|  |  | | Constant | -5.711 | 1.30 | -4.36 |  | -6.963 | 1.692 | -4.11 |
|  |  | | Α | 1.217 | 0.330 |  |  | 3.778 | 0.638 |  |
|  | | N=294 | |  |  |  |  |  |  |  |
