# Supplementary figures and images for: Using social media to promote academic research: Identifying the benefits of twitter for sharing academic work
Source: PLoS One. 2020 Apr 6;15(4):e0229446. doi: 10.1371/journal.pone.0229446 (PMC7135289; doi:10.1371/journal.pone.0229446)

# **S4 Appendix. Distribution of Counts of Tweets**

# **
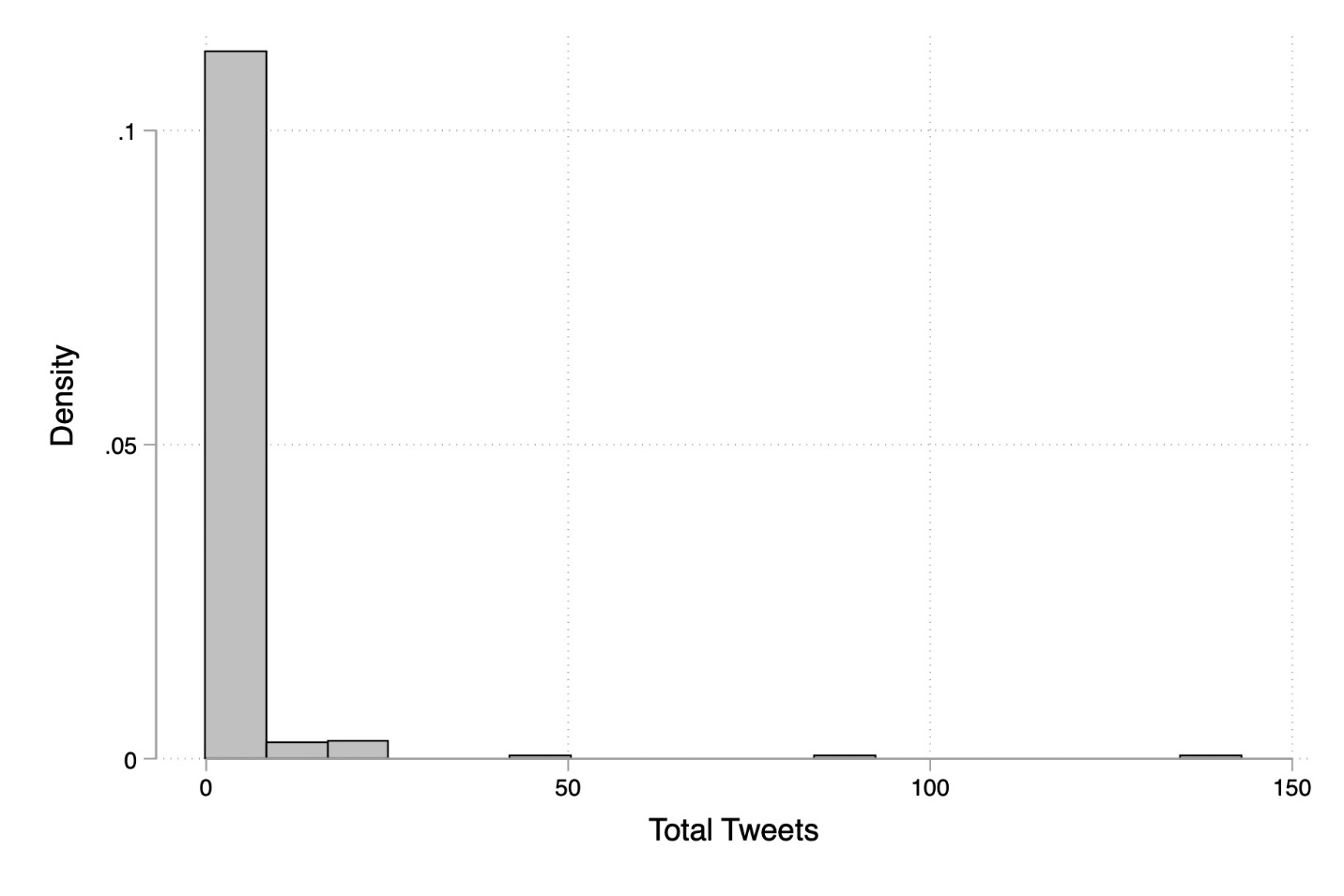
**

Supplement: S4 Appendix — (DOCX) [file pone.0229446.s004.docx]
