# Supplementary material for: Using social media to promote academic research: Identifying the benefits of twitter for sharing academic work
Source: PLoS One. 2020 Apr 6;15(4):e0229446. doi: 10.1371/journal.pone.0229446 (PMC7135289; doi:10.1371/journal.pone.0229446)
Supplement: S5 Appendix — a. Tweet content coding. b. Results of content coding, by author gender. (DOCX) [file pone.0229446.s005.docx]

# **S5 Appendix. a. Tweet Content Coding.**

The content of the originating tweet was coded in two ways, first along the categories listed in Table A5a1 and second in categories listed in Table A5a2 (which separates self-promotion tweets from tweets made by someone who is not the article’s author). The third column of each table represents an example of a tweet from the sample that was coded in this way. Tweets that were not in English were translated for coding.

| Table A5a1. Article Content Coding (Version 1) | | |
| --- | --- | --- |
|  | Content | Example Tweet |
| 1 | Article title and link only | “Can Employment Reduce Lawlessness and Rebellion? A Field Experiment with High-Risk Men in a Fragile State http:// ow.ly/JkNY30eDZ8T” |
| 2 | Link and a statement noting that the paper is newly published/available. | “The APSR article @johannarickne and I wrote on gender quotas and women's political leadership is ungated thru Aug. http:// bit.ly/2uaPSwl” |
| 3 | Link and a statement offering the paper as instructive (i.e. helpful method, helpful to someone’s question) | “pay at least as much attention on the theoretical contribution than on the design/method. On examples: here is one based on field research https:// doi.org/10.1017/S00030 55416000277 … 4/” |
| 4 | Link and a statement of the paper’s argument. | “Well being related to distance from and exposure to the official language https://www. cambridge.org/core/journals/ american-political-science-review/article/language-policy-and-human-development/F0464F77F597CA8FA466758650718018 …” |
| 5 | Link and critique of the paper. | None |
| 6 | Link and a comment that cannot fit into other categories. | “Hi, do you know why I can't access the supplementary material to this paper? Thanks! https://www. cambridge.org/core/journals/ american-political-science-review/article/explaining-causal-findings-without-bias-detecting-and-assessing-direct-effects/D11BEB8666E913A0DCD7D0B9872F5D11#fndtn-supplementary-materials …” |

| Table A5a2. Article Content Coding (Version 2) | | |
| --- | --- | --- |
|  | Content | Example Tweet |
| 1 | Article title and link only | “Can Employment Reduce Lawlessness and Rebellion? A Field Experiment with High-Risk Men in a Fragile State http:// ow.ly/JkNY30eDZ8T” |
| 2 | Link and a statement noting that the paper is newly published/available (not by the author) | “Some of my favorite work on quotas by @dianazobrien & @johannarickne is currently ungated! Read it! https://www. cambridge.org/core/journals/ american-political-science-review/article/gender-quotas-and-womens-political-leadership/0C8CC7B51CC37871A1B1FD925A8AA976 …” |
| 3 | Link and a statement offering the paper as instructive (i.e. helpful method, helpful to someone’s question) | “pay at least as much attention on the theoretical contribution than on the design/method. On examples: here is one based on field research https:// doi.org/10.1017/S00030 55416000277 … 4/” |
| 4 | Link and a statement of the paper’s argument. | “Well being related to distance from and exposure to the official language https://www. cambridge.org/core/journals/ american-political-science-review/article/language-policy-and-human-development/F0464F77F597CA8FA466758650718018 …” |
| 5 | Link and critique of the paper. | None |
| 6 | Link and a comment that cannot fit into other categories. | “Hi, do you know why I can't access the supplementary material to this paper? Thanks! https://www. cambridge.org/core/journals/ american-political-science-review/article/explaining-causal-findings-without-bias-detecting-and-assessing-direct-effects/D11BEB8666E913A0DCD7D0B9872F5D11#fndtn-supplementary-materials …” |
| *7* | *Self-promotion* | *“The APSR article @johannarickne and I wrote on gender quotas and women's political leadership is ungated thru Aug. http:// bit.ly/2uaPSwl”* |

**S5 Appendix. b. Results of content coding, by author gender.**

| Table A5b1: Results of Coding Content | | | |
| --- | --- | --- | --- |
| Content code | Overall | Woman author | No woman author |
| Article title and link only | 53.68% | 53.01% | 54.21% |
| Link and a statement noting that the paper is newly published/available. | 6.84% | 10.84% | 3.74% |
| Link and a statement offering the paper as instructive (i.e. helpful method, helpful to someone’s question) | 9.47% | 8.43% | 10.28% |
| Link and a statement of the paper’s argument. | 26.32% | 22.89% | 28.97% |
| Link and critique of the paper. | 0 | 0 | 0 |
| Link and a comment that cannot fit into other categories. | 3.16% | 3.61% | 2.80% |

| Coding Version 2 (separates out self-promotion) |  |  |  |
| --- | --- | --- | --- |
| Content code | Overall | Woman author | No woman author |
| Article title and link only | 53.16% | 51.81% | 54.21% |
| Link and a statement noting that the paper is newly published/available. | 4.74% | 8.43% | 1.87% |
| Link and a statement offering the paper as instructive (i.e. helpful method, helpful to someone’s question) | 8.95% | 7.23% | 10.28% |
| Link and a statement of the paper’s argument. | 22.63% | 18.07% | 26.17% |
| Link and critique of the paper. | 0 | 0 | 0 |
| Link and a comment that cannot fit into other categories. | 3.16% | 3.61% | 2.80% |
| Tweeting about self | 6.84% | 9.64% | 4.67% |
