# Supplementary material for: Using social media to promote academic research: Identifying the benefits of twitter for sharing academic work
Source: PLoS One. 2020 Apr 6;15(4):e0229446. doi: 10.1371/journal.pone.0229446 (PMC7135289; doi:10.1371/journal.pone.0229446)
Supplement: S6 Appendix — The figure includes some jitter because there are multiple articles with 0 citations at both time points. (DOCX) [file pone.0229446.s006.docx]

**S6 Appendix. Relationship between Citations in 2018 and 2019. The figure includes some jitter because there are multiple articles with 0 citations at both time points.**


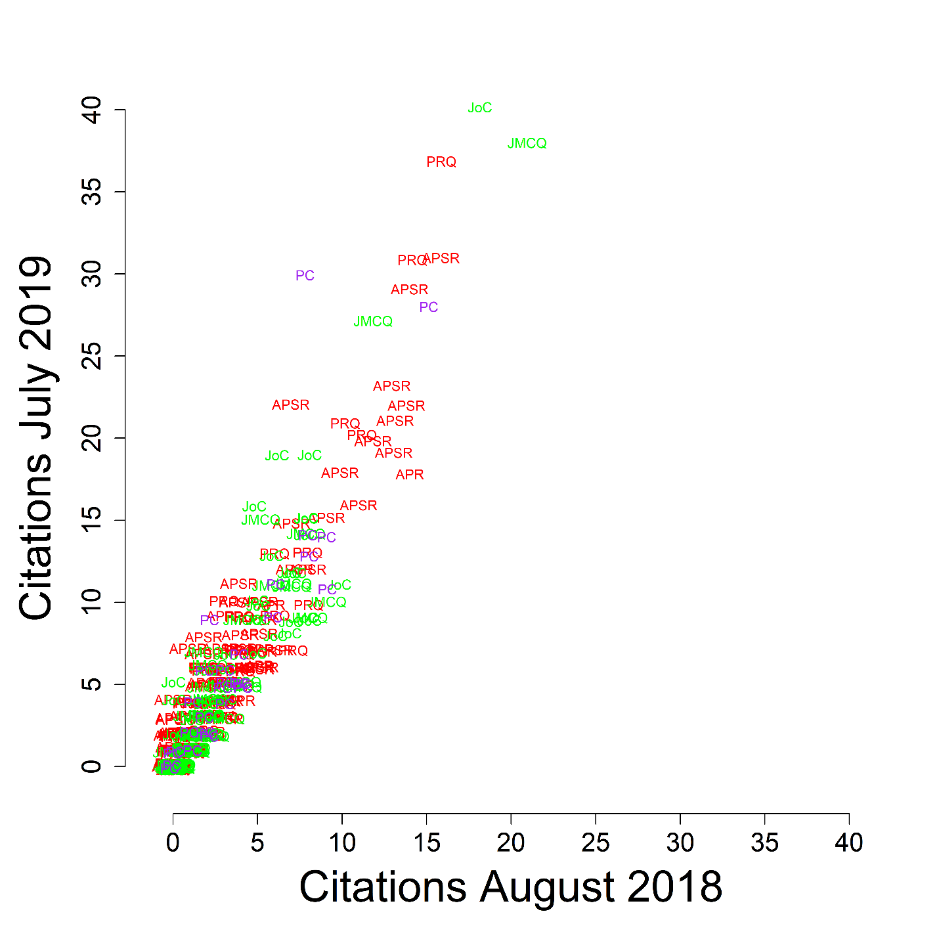


*Each point represents an article; the journal title where the article is published is used to denote a point.*
