# Supplementary material for: Using social media to promote academic research: Identifying the benefits of twitter for sharing academic work
Source: PLoS One. 2020 Apr 6;15(4):e0229446. doi: 10.1371/journal.pone.0229446 (PMC7135289; doi:10.1371/journal.pone.0229446)
Supplement: S7 Appendix — (DOCX) [file pone.0229446.s007.docx]

**S7 Appendix. Predicting the number of citations by article characteristics and tweets, excluding outlier.**

|  |  | 2018 Citations | |
| --- | --- | --- | --- |
|  |  | Coef. | S.E. |
|  | Citations 2018 |  |  |
|  | Any Tweets | 0.681 | 0.170 |
|  | Total Tweets | 0.001 | 0.009 |
| *Author Information* | |  |  |
|  | % of Women | -0.712 | 0.009 |
|  | Number of Authors | 0.009 | 0.094 |
|  | % Women X Number of Authors | 0.427 | 0.190 |
|  | Logged Twitter Followers | 0.032 | 0.019 |
|  | Mean Academic Rank of Authors | 0.049 | 0.095 |
| *Article Subfield* | |  |  |
|  | International Relations | -0.536 | 0.575 |
|  | Comparative Politics | -0.136 | 0.546 |
|  | Political Philosophy | -1.171 | 0.581 |
|  | American Politics | -0.415 | 0.546 |
|  | Communications | -0.571 | 0.609 |
| *Journal* | |  |  |
|  | Journal of Communication | 0.602 | 0,510 |
|  | JMCQ | 0.552 | 0.495 |
|  | Political Communication | 0.180 | 0.284 |
|  | Political Research Quarterly | 0.184 | 0.283 |
|  | APSR | 0.830 | 0.283 |
|  | Constant | 1.19 | 0.676 |
| Α | | 0.855 | 0.095 |
| AIC | | 1605.176 | |
| N | | 293 | |
