# Supplementary material for: Using social media to promote academic research: Identifying the benefits of twitter for sharing academic work
Source: PLoS One. 2020 Apr 6;15(4):e0229446. doi: 10.1371/journal.pone.0229446 (PMC7135289; doi:10.1371/journal.pone.0229446)
Supplement: S8 Appendix — (DOCX) [file pone.0229446.s008.docx]

**S8 Appendix. Modelling the effect of author gender on citations without controlling for the number of tweets.**

|  |  | Model 1 | | Model 2 | |
| --- | --- | --- | --- | --- | --- |
|  |  | Coef. | S.E. | Coef. | S.E. |
| *Author Information* | |  |  |  |  |
|  | % of Women | 0.110 | 0.160 | -0.193 | 0.343 |
|  | Number of Authors | 0.186 | 0.072 | 0.127 | 0.092 |
|  | % Women X Number of Authors | - | - | 0.188 | 0.190 |
|  | Logged Twitter Followers | 0.039 | 0.020 | 0.038 | 0.020 |
|  | Mean Academic Rank of Authors | 0.070 | 0.099 | 0.070 | 0.099 |
| *Article Subfield* | |  |  |  |  |
|  | International Relations | -0.425 | 0.601 | -0.498 | 0.603 |
|  | Comparative Politics | -0.031 | 0.570 | -0.082 | 0.569 |
|  | Political Philosophy | -1.165 | 0.604 | -1.254 | 0.608 |
|  | American Politics | -0.184 | 0.571 | -0.248 | 0.573 |
|  | Communications | -0.323 | 0.601 | -0.472 | 0.645 |
| *Journal* | |  |  |  |  |
|  | Journal of Communication | 0.739 | 0.543 | 0.703 | 0.544 |
|  | JMCQ | 0.749 | 0.528 | 0.741 | 0.527 |
|  | Political Communication | 0.768 | 0.272 | 0.780 | 0.272 |
|  | Political Research Quarterly | 0.505 | 0.253 | 0.516 | 0.253 |
|  | APSR | 1.241 | 0.284 | 1.254 | 0.285 |
|  | Constant | 0.581 | 0.684 | 1.223 | 0.679 |
| Α | | 0.961 | 0.102 | 0.958 | 0.102 |
| AIC | | 1632.264 | | 1633.296 | |
| N | | 293 | | 294 | |
