# Supplementary material for: Using social media to promote academic research: Identifying the benefits of twitter for sharing academic work
Source: PLoS One. 2020 Apr 6;15(4):e0229446. doi: 10.1371/journal.pone.0229446 (PMC7135289; doi:10.1371/journal.pone.0229446)
Supplement: S1 File — (PDF) [file pone.0229446.s009.pdf]

Klar, Samara M - (klar)

From: Burchfield, Mason L - (mlburchfi)
Sent: Tuesday, December 3, 2019 12:58 PM
To: Klar, Samara M - (klar)
Subject: RE: Following up on question re: publicly available Twitter handles
Attachments: determination_v2019-08-15 (4).pdf

Hi Samara,
Thanks for your call!

Your understanding is correct. According to our regulations, Public use Datasets are not considered Human Subjects Research. A Public Dataset is defined as an activity that is limited to analyzing information contained within a publically available dataset (Meaning, any person can find and use the data). In this case, because the twitter handles are public, your activity would not fall under IRB purview.

If you'd like an official letter of determination, you can complete the attached "Determination of Human Research" form and submit it to our department inbox at vpr-irb@email.arizona.edu. This is not a necessary step.

Please let me know if you have any questions.

Thank you,
Mason L. Burchfield, CIP
IRB Coordinator
Human Subjects Protection Program
The University of Arizona
1618 E. Helen Street
PO Box 245137
office: 520-626-0433
mlburchfi@email.arizona.edu

Let us know how we are doing through the following website survey:
https://uarizona.co1.qualtrics.com/jfe/form/SV_dgQSVxqciPhiiUd.

From: Klar, Samara M - (klar) <klar@email.arizona.edu>
Sent: Tuesday, December 3, 2019 12:31 PM
To: Burchfield, Mason L - (mlburchfi) <mlburchfi@email.arizona.edu>
Subject: Following up on question re: publicly available Twitter handles

Hi Mason,

We just spoke about a question I had regarding publishing publicly available Twitter handles. I just wanted to follow up to make sure that I understood correctly. If the twitter handles are publicly available, they are not under the purview of the IRB, is that correct? If you could just explain again so I know I'm following, I'd really appreciate that!

Many thanks!
Samara

**

Samara Klar, Ph.D.
Associate Professor
School of Government & Public Policy
University of Arizona
Tucson, Arizona

Call/Text: 262-372-6726
Email: klar@email.arizona.edu
Web: www.samaraklar.com
