# Supplementary material for: Using social media to promote academic research: Identifying the benefits of twitter for sharing academic work
Source: PLoS One. 2020 Apr 6;15(4):e0229446. doi: 10.1371/journal.pone.0229446 (PMC7135289; doi:10.1371/journal.pone.0229446)
Supplement: S5 File — (PDF) [file pone.0229446.s013.pdf]

Klar, Samara M - (klar)

**From:**	Yosuke SUNAHARA <sunaharay@gmail.com>
**Sent:**	Tuesday, December 3, 2019 4:44 PM
**To:**	Klar, Samara M - (klar)
**Subject:**	Re: Seeking permission to include your twitter handle in a table

Dear Dr. Klar,

Thank you for letting me know about your project. It seems to be very interesting, and I don't find any problems when you use my tweets, which are open to the public.
Definitely, I myself get the information about journal articles through Twitter and frequently cite them in my works. But, I'm not sure my tweets contribute to spreading peer-reviewed articles because most of my followers are interested in the news in Japanese...

Anyway, I hope your work is going well and look forward to reading the published one!

All the best,

Yosuke

2019年12月4日(水) 5:26 Klar, Samara M - (klar) <klar@email.arizona.edu>:

> Dear Dr. Sunahara,
>
> I'm writing to ask for your consent to include your twitter handle in an article I am coauthoring (along with Yanna Krupnikov, John Ryan, Kathleen Searles, and Yotam Shmargad).
>
> In our article, we analyze Twitter activity among academics. We are particularly interested in exploring whether journal articles that are shared on twitter tend to be cited more than those that are not. We carry out our analyses in a number of ways and we do find modest effects: that is, articles that are shared on twitter appear to be cited more often – even a year later – than similar articles that are not. We also find no discrepancies by gender.
>
> Our dataset includes publicly available Twitter handles of academics who shared peer-reviewed articles during the time-period we examined. Your handle is among them. According to the IRB, these handles are publicly available and thus do not require consent to publish. However, we still wanted to reach out to obtain your consent for including your Twitter handle in a table of academics who post articles on Twitter. This article is currently R+R'ed at Plos ONE and, should it be accepted, your handle would be included in a table in the article.

Please let me know if you are okay with this! I am also happy to answer any questions or concerns you might have!

Best,

Samara

**

Samara Klar, Ph.D.
Associate Professor

School of Government & Public Policy

University of Arizona

Tucson, Arizona

Call/Text: 262-372-6726

Email: klar@email.arizona.edu

Web: www.samaraklar.com

--
SUNAHARA, Yosuke, Ph.D.
Professor of Public Administration
Graduate School of Law, Kobe University
Tel / Fax. 078-803-6774
mailto : sunaharay@gmail.com / sunahara@people.kobe-u.ac.jp
website: http://www.geocities.jp/yosuke_sunahara/
